# Inhibitors of both the *N*-methyl lysyl- and arginyl-demethylase activities of the JmjC oxygenases

**DOI:** 10.1098/rstb.2017.0071

**Published:** 2018-04-23

**Authors:** Joanna Bonnici, Anthony Tumber, Akane Kawamura, Christopher J. Schofield

**Affiliations:** 1 Department of Chemistry, Chemistry Research Laboratory, University of Oxford, Mansfield Road, Oxford OX1 3TA, UK; 2 Division of Cardiovascular Medicine, Radcliffe Department of Medicine, Wellcome Trust Centre for Human Genetics, University of Oxford, Roosevelt Drive, Oxford OX3 7BN, UK

**Keywords:** histone demethylases, epigenetics, lysine and arginine demethylation, 2-oxoglutarate/α-ketoglutarate oxygenases, JmjC proteins

## Abstract

The Jumonji C (JmjC) family of 2-oxoglutarate (2OG)-dependent oxygenases have established roles in the regulation of transcription via the catalysis of demethylation of *N*ε*-*methylated lysine residues in histone tails, especially the N*-*terminal tail of histone H3. Most human JmjC *N*^*ɛ*^-methyl lysine demethylases (KDMs) are complex enzymes, with ‘reader domains’ in addition to their catalytic domains. Recent biochemical evidence has shown that some, but not all, JmjC KDMs also have *N*ω*-*methyl arginyl demethylase (RDM) activity. JmjC KDM activity has been linked to multiple cancers and some JmjC proteins are therapeutic targets. It is, therefore, important to test not only whether compounds in development inhibit the KDM activity of targeted JmjC demethylases, but also whether they inhibit other activities of these proteins. Here we report biochemical studies on the potential dual inhibition of JmjC KDM and RDM activities using a model JmjC demethylase, KDM4E (JMJD2E). The results reveal that all of the tested compounds inhibit both the KDM and RDM activities, raising questions about the *in vivo* effects of the inhibitors.

This article is part of a discussion meeting issue ‘Frontiers in epigenetic chemical biology’.

## 1. Background

**Figure 1.**
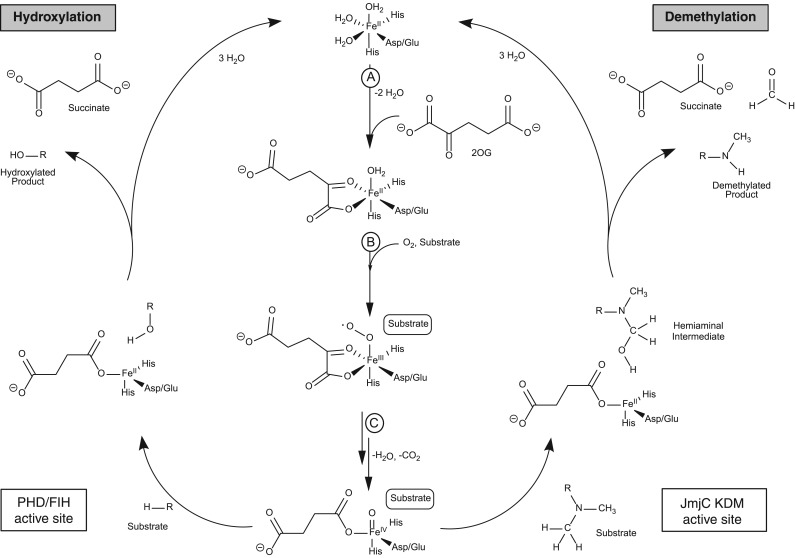
Proposed catalytic 2OG-dependent mechanism as carried out by ‘hydroxylases’ (e.g. the prolyl hydroxylase domain-containing protein enzymes (PHDs) and factor inhibiting 2OG-dependent hypoxia inducible factor (FIH)) and demethylases (i.e. the JmjC KDM2-7 human subfamilies). The figure is adapted from [1,2]. Methylated substrates of the JmjC KDMs include mono-, di-, and tri-methylated lysine residues and mono- and di-methylated guanidino nitrogens of arginine resides. In the case of ‘hydroxylases’ (e.g. PHDs and FIH) substrates include 2OG-dependent hypoxia inducible factor (HIF) prolyl- and asparaginyl-residues in HIF-α; FIH also accepts multiple ankyrin repeat domain proteins. Steps A to C are common to both types of 2OG oxygenases. (A) 2OG binds to the active site Fe(II) in a bidentate manner with consequent loss of two water molecules. (B) Substrate binding is followed by O_2_ binding (with displacement of H_2_O), resulting in, (C), oxidative decarboxylation of 2OG to give succinate, CO_2,_ and an Fe(IV)=O intermediate. PHD/FIH active site (hydroxylation) involves formation of a stable alcohol product. JmjC KDM/RDM (demethylation) involves hydroxylation of the *N*-methyl group to give a hemiaminal that fragments to produce formaldehyde and the demethylated product.

In organisms varying from bacteria to humans, 2-oxoglutarate (2OG) and Fe(II)-dependent oxygenases play important roles in the regulation of gene expression and protein biosynthesis (figure 1) [1–3]. In humans, and other eukaryotes, such roles include the regulation of transcription via the modification of nucleic acids, histones and transcription factors [4]. The 2OG-dependent hypoxia inducible factor (HIF) hydroxylases were the first such enzymes shown to have a role in transcriptional regulation. HIF is an α/β-heterodimeric transcription factor that upregulates the transcription of hundreds of genes, which collectively work to ameliorate the effects of hypoxia (i.e. by limiting oxygen availability) [5]. Prolyl-hydroxylation of the HIF-α subunit, which is catalysed by the 2OG-dependent prolyl hydroxylase domain-containing protein enzymes (PHDs), signals for HIF degradation via the ubiquitin protease system. There is substantial evidence that PHD catalysis is regulated by oxygen availability in cells and that it acts as a hypoxia-sensing mechanism for the HIF system. PHD inhibitors are at late stage clinical trials for the treatment of anaemia, because erythropoietin is a HIF target gene [1].

A second type of 2OG oxygenase-dependent HIF-α modification is catalysed by factor inhibiting HIF (FIH), which also regulates HIF activity. In humans, and in other higher animals, FIH catalyses the hydroxylation of an asparaginyl-residue in the C-terminal transactivation domain of HIF-α subunits—a post-transcriptional modification that reduces the binding of HIF-α subunits to transcriptional co-activator p300-CBP family of histone acetyl transferases [6]. The two types of HIF hydroxylases belong to different structural subfamilies of 2OG oxygenases, with the PHDs belonging to the deacetoxycephalosporin C synthase subfamily, which normally have relatively simple domain architectures [7]. By contrast, FIH belongs to the normally multidomain JmjC-containing family of protein [8,9]. Subsequent work identified other subtypes of JmjC oxygenases [10–14]. FIH is one of the JmjC ‘hydroxylases’ that catalyse the formation of stable alcohol products [6,15]. Such enzymes include the ribosomal/protein oxygenases and related 2OG oxygenases catalysing modification of the translational machinery [15–17] (figure 1).

*Nɛ*-methyl lysine demethylases (KDMs) form a subfamily of JmjC oxygenases that is highly conserved in eukaryotes. The JmjC KDMs likely evolved from the JmjC hydroxylases [18] and form the largest known family of KDMs, with the flavin-dependent lysine specific demethylases (LSDs) being less ubiquitous [18,19]. The JmjC KDMs catalyse demethylation of all *Nɛ*-methylation states of lysine residues (figure 1), whereas the LSDs only accept the di- and mono-*Nɛ*-methylation states [6,14,15]. There are six human subfamilies of JmjC KDMs that act on histone tails with different *Nɛ*-methylation states, histone sequence and ‘adjunct’ protein selectivities. The JmjC KDMs play central roles in modulating the dynamic post-translational methylation states of histones and can regulate transcription [14,19–21]. The JmjC KDMs have been linked to multiple diseases, including mutations that correlate with genetic diseases [22]. Some KDMs are involved in proliferation and tumour growth, and some are overexpressed in various cancer types, including breast cancer and prostate cancer [12,23,24]. As a consequence, certain JmjC KDMs, including the KDM4 and KDM5 subfamilies, are being pursued as medicinal chemistry targets.

The correct assignment of function at the (patho)physiological level will be important in the rational development of 2OG oxygenase inhibitors. One clinically used compound, meldonium (used to treat myocardial infarction), is proposed to target a human 2OG oxygenase, γ-butyrobetaine hydroxylase, but the precise molecular mode of action of meldonium is uncertain [25,26]. In the case of HIF, a combination of biochemical and physiological studies led to the proposal of an unexpectedly direct connection between the reaction catalysed by the PHDs (prolyl-hydroxylation of HIF-α, figure 1) and hypoxia sensing in animals [27,28], a finding that has stimulated the clinical development of PHD inhibitors for treatment of anaemia and other hypoxia-related diseases [1]. Subsequent work has revealed complexities in the HIF system, including reports of a number of non-HIF-α PHD substrates; however, the biological relevance of these substrates and their involvement in the hypoxic response are unclear, and the key role of the PHDs in HIF-mediated hypoxic response appears robust [21,29,30]. In contrast, there is good evidence that FIH not only accepts multiple non-HIF substrates—many from the ankyrin repeat domains (ARD) structural family—but it can also catalyse the hydroxylation of residues other than asparagine, including aspartyl- and histidyl-residues [21,31]. Like the proposed non-HIF PHD substrates, the biological roles of FIH-catalysed ARD hydroxylation are unclear, but they may serve to regulate the availability of FIH for HIF-α hydroxylation [21,31]. The promiscuous nature of FIH, in terms of its substrate and product selectivity, echoes that of 2OG oxygenases and structurally related enzymes involved in biosynthesis [4,21].

We have been exploring the substrate/product selectivities of the assigned JmjC KDMs. We have found that some of them will not only accept a number of unnatural substrate analogues, but also catalyse *N*ω-methyl arginine residue demethylation (RDM), albeit with the RDM activity being lower than the KDM activity [32,33]. The RDM activity of the JmjC demethylases has not been validated in cells, in part due to the paucity of appropriate reagents and antibodies and, in part, due to the complexity of the chemistry of the post-translational modifications on the histone H3 tail [32,33]. However, the finding that the JmjC demethylases can catalyse *N*ω-methyl arginine residue demethylation raises questions on the mode of action of the JmjC demethylase inhibitors that are in development for use as cancer drugs. As with all the HIF PHD inhibitors that are in clinical trials [1], most (but not all) of the JmjC KDM inhibitors that have been developed compete with 2OG and complex to the active site-bound Fe(II) (figure 1) [34]. Further, clinically used iron-chelating compounds such as deferoxamine and ethylenediaminetetraacetic acid (EDTA) have potential to inhibit both the KDM and RDM activity of the JmjC demethylases. Here we report studies that compare the inhibition of KDM and RDM activity by a set of reported JmjC demethylase inhibitors with the model enzyme KDM4E (JMJD2E). The results reveal that the JmjC KDM inhibitors also inhibit RDM activity of KDM4E, thus raising questions regarding the mode(s) of action of the inhibitors in the cellular context.

## 2. Results

To investigate whether JmjC KDM inhibitors also inhibit RDM activity, we elected to work with KDM4E, a likely pseudo gene in humans. KDM4E is a relatively simple model JmjC enzyme, which is reported to have both KDM and RDM activities and which can be readily produced in recombinant form [32]. The KDM4D, E and F genes are located on a single exon and encode for only Jumonji N (JmjN) and JmjC domains. By contrast, the genes for the other human members of the KDM4 family, i.e. A, B and C, are located on multiple exons and encode for JmjN, JmjC, PHD-fingers and two TUDOR domains [35,36].

**Figure 2.**
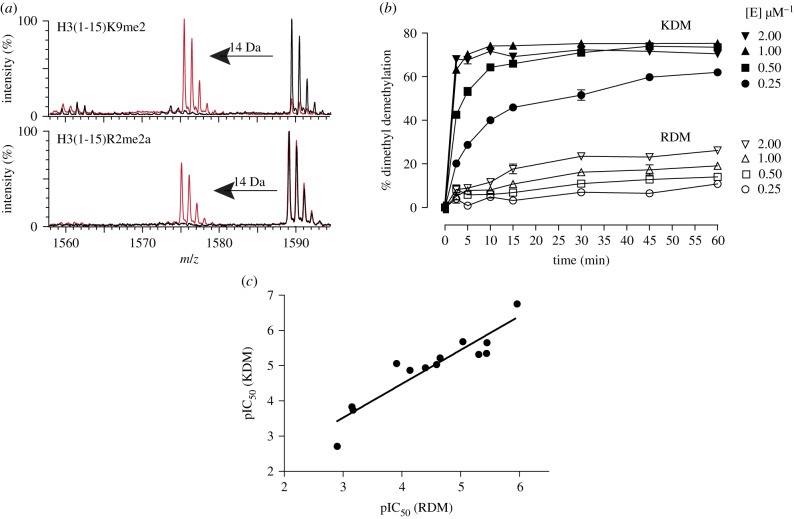
(*a*) Matrix assisted laserd desorption/ionization - time of flight mass spectrometry (MALDI-TOF MS) showing demethylation of H3(1-15)K9me2 (top) and H3(1-15)R2me2a (bottom) as catalysed by KDM4E. A −14 Da shift can be observed after 15 min for both substrates, indicating loss of a methyl group*.* (*b*) MALDI-TOF MS time-course assays showing the fraction of demethylation by KDM4E. Error bars represent s.d. for analytical replicates, *n* = 2/3*.* Conditions: 10 µM respective peptide, 10 µM (NH_4_)_2_Fe(SO_4_)_2_, 100 µM sodium l-ascorbate, 10 µM 2OG. (*c*) Correlation for KDM4E KDM versus RDM pIC_50_ values.

Recombinant KDM4E was produced in *Escherichia coli* and purified as reported [37]. We initially optimized assay conditions for comparing KDM/RDM activities using 15mer H3 peptide fragments. These either had a dimethylated lysine at position nine (H3(1-15)K9me2) or asymmetrically dimethylated arginine at position two (H3(1-15)R2me2a) (figure 2). Kinetic analysis of KDM4E-catalysed demethylation of H3(1-15)K9me2 and H3(1-15)R2me2a peptides confirmed that lysine demethylation was more rapid than arginine demethylation, with specific activities of 13 nmole min^−1^ mg^−1^ for H3(1-15)K9me2 and 0.3 nmole min^−1^ mg^−1^ for H3(1-15)R2me2a demethylation under the employed assay conditions (figure 2 and electronic supplementary material, S1). These observations are consistent with the catalytic efficiencies of these peptides (*k*_cat_/*K*_M_ = 114 × 10^−6^ s^−1^ µM^−1^ for H3(1-15)R2me2a and 2167.2 × 10^−6^ s^−1^ µM^−1^ for H3(1-15)K9me2) (electronic supplementary material, table S1). We then tested various classes of reported JmjC KDM inhibitors against both the KDM and RDM activities, using RapidFire Mass Spectrometry (RF-MS), a high throughput-based MS assay. To ensure robust results, the KDM and RDM assays differed in their endpoints (15 and 25 min, for KDM and RDM assays, respectively) and also in the enzyme concentrations used (0.25 and 1 µM for KDM and RDM assays, respectively). The 2OG concentration was 10 µM, its approximate *K*_M_ value (electronic supplementary material, table S1). A panel of JmjC KDMs inhibitors were then selected (see electronic supplementary material, figure S2 for compound structures) and tested under the optimized KDM and RDM assay conditions.

Initially, we tested the broad-spectrum 2OG oxygenase inhibitors 5-carboxy-8-hydroxyquinoline (IOX1), 2,4-pyridinedicarboxylic acid (2,4-PDCA) and *N*-oxalylglycine (NOG) [38–41]. Both RDM and KDM activities were inhibited approximately equally by IOX1 (pIC_50_ (RDM) = 5.45 ± 0.03; pIC_50_ (KDM) = 5.65 ± 0.03), while 2,4-PDCA (pIC_50_ (RDM) = 5.96 ± 0.03 and pIC_50_ (KDM) = 6.75 ± 0.02) and NOG (pIC_50_ (RDM) = 3.91 ± 0.10; pIC_50_ (KDM) = 5.06 ± 0.05) showed somewhat better inhibition of KDM4E KDM activity. IOX1, 2,4-PDCA and NOG are 2OG competitive inhibitors [39–41], whereas IOX1 has been reported to exhibit mixed-mode inhibition and to cause movement of the active site metal [38] (table 1, electronic supplementary material, figure S2). Note that care should be taken in drawing conclusions from (small) differences between KDM and RDM potencies due to the different assay conditions.

**Table 1.** pIC_50_ values for various inhibitors against KDM4E KDM and RDM activity. Dose–response experiments were performed using the RF-MS-based assay (*n* = 3 assay repeats, each assay replicate has *n* = 3 analytical replicates). Conditions: 5 µM H3(1-15)K9me2/H3(1-15)R2me2a with 0.25 µM/1 µM KDM4E, respectively, 10 µM (NH_4_)_2_Fe(SO_4_)_2_, 100 µM sodium l-ascorbate, 10 µM 2OG; *t* = 15/25 min for KDM and RDM activities, respectively, following from analyses shown in electronic supplementary material, figure S1. Structures of inhibitors are given in electronic supplementary material, figure S2. s.e.m., standard error of mean.

inhibitor	pIC_50_ ± s.e.m.	
	inhibition of H3(1-15)K9me2 demethylation	inhibition of H3(1-15)R2me2a demethylation
IOX1	5.65 ± 0.03	5.45 ± 0.03
2,4-PDCA	6.75 ± 0.02	5.96 ± 0.03
NOG	5.06 ± 0.05	3.91 ± 0.10
deferoxamine^a^	5.62 ± 0.03	5.45 ± 0.06
EDTA^a^	5.82 ± 6.53	5.95 ± 0.06
CPI-455	5.35 ± 0.03	5.44 ± 0.04
KDOAM25	3.74 ± 0.14	3.17 ± 0.34
KDM5-C49	5.68 ± 0.04	5.04 ± 0.05
SD70	5.22 ± 0.02	4.65 ± 0.04
ML324	5.32 ± 0.02	5.31 ± 0.04
JIB-04	5.03 ± 0.03	4.59 ± 0.07
Ni(II)^a^	6.84 ± 0.03	5.80 ± 0.06
Co(II)^a^	7.17 ± 0.02	6.19 ± 0.04
fumarate^a^	2.71 ± 0.05	2.90 ± 0.07
succinate^a^	3.83 ± 0.04	3.15 ± 0.05
l-2HG^a^	4.87 ± 0.03	4.14 ± 0.05
d-2HG^a^	4.94 ± 0.02	4.40 ± 0.05

^a^*n* = 2 assay repeats.

We then tested the iron chelators Deferoxamine and EDTA. Both chelators inhibited both KDM and RDM activities with similar pIC_50_ values (deferoxamine: pIC_50_ (RDM) = 5.45 ± 0.06 and pIC_50_ (KDM) = 5.62 ± 0.03, EDTA: pIC_50_ (RDM) = 5.95 ± 0.06, pIC_50_ (KDM) = 5.82 ± 6.53) (table 1, electronic supplementary material, figure S2). Nickel(II) and cobalt(II) ions also inhibited both KDM and RDM activities, likely via competition with Fe(II) for binding [42].

Certain TCA cycle metabolites inhibit 2OG oxygenases, including the JmjC KDMs with varying potencies [37,39,43,44]. In addition, levels of both enantiomers of 2-hydroxyglutarate (*l*-2HG and *d-*2HG) can be substantially increased in some tumours [45]. Succinate, fumarate, *l*-2HG and *d-*2HG were thus tested for inhibition against the KDM and RDM activities of KDM4E. These metabolites inhibited both KDM and RDM activities, consistent with prior reports on JmjC KDM inhibition by 2-hydroxyglutarate [39,44] (table 1, electronic supplementary material, figure S2). Notably, both enantiomers of 2-hydroxyglutarate inhibited KDM and RDM activities with similar efficiency, which accords with their reported inhibition of the KDM activities of KDM4A and KDM4C (but not of all 2OG oxygenases) [39].

Inhibitors reported to be selective for specific JmjC KDM subfamilies were then tested [34]. CPI-455, KDOAM25 and KDM5-C49 are all reported to be selective inhibitors of the KDM5 subfamily [46–48]. For both CPI-455 and KDM5-C49, which are reported 2OG competitors [46,48], both KDM and RDM activities of KDM4E were inhibited (in accordance with the reported values for KDM4C [46,48]), with pIC_50_ values within a close range. As expected from its reported selectivity profile [47], KDOAM25 proved to be the weakest KDM4E KDM and RDM inhibitor with pIC_50_ < 4, further supporting its selectivity towards the KDM5 subfamily. SD70, ML324 and JIB-04 (which unusually to date is identified as a non 2OG competitor [49]) have been reported to be selective inhibitors of the KDM4 subfamily [49–51]. All these compounds inhibited both KDM and RDM activities and in each case (except for ML324) with a slightly lower value for the RDM activity, but all comparable with published IC_50_ (KDM) values. ML324 was an equipotent inhibitor for the KDM and RDM activities (table 1 and electronic supplementary material, figure S2).

Analysis of KDM and RDM pIC_50_ values showed a significant correlation (Spearman rank correlation *ρ* = 0.86, Pearson's *r* = 0.93, *p*-value < 0.0001), with no significant outliers (figure 2). Thus, the overall results reveal that the analysed compounds inhibit both KDM and RDM KDM4E activities, i.e. are indiscriminate whether the substrate is H3(1-15)K9me2 or H3(1-15)R2me2a.

## 3. Discussion

The results demonstrate that all of the tested JmjC KDM inhibitors that inhibit via metal chelation (2OG mimetics/generic metal chelators) inhibit both the KDM and RDM activities of KDM4E. Overall, the potencies of inhibition were similar for the KDM and RDM activities, although in some cases, there were small differences in the apparent potencies. These differences are likely (at least in part) to be due to the different assay conditions for the KDM and RDM activities, namely, the enzyme concentrations used due to KDM4E catalytic efficiency being 20-fold higher for the H3(1-15)K9me2 peptide than for the H3(1-15)R2me2a peptide (electronic supplementary material, figure S1 and table S1). Although KDM4E is a model JmjC KDM, it is likely that the KDM/RDM dual inhibition will be observed for most, if not all, JmjC demethylases with RDM activity. This is because of the overall similarities in the active sites of the JmjC demethylases and the fact that all tested inhibitors work either by chelation with the active site iron or by chelating iron in solution [2]. Although the RDM activity of the JmjC KDMs has not been verified in a cellular context, the results clearly highlight the need to consider potential activities beyond KDM catalysis when considering the effects of JmjC demethylase inhibitors, whether in pharmaceutical form or when produced endogenously, as is the case as a consequence of TCA cycle enzyme-associated mutations [37,39,43].

Given the apparent promiscuity of at least some JmjC demethylases [33], activities beyond KDM/RDM catalysis should also not be ruled out. In this regard, it is notable that one other human 2OG oxygenase, JMJD6, has also been reported to have RDM activity [52]. However, subsequent work revealed that JMJD6 catalyses hydroxylation of lysyl-C-5 of mRNA splicing factor proteins and of histones [53,54]. JmjC demethylases may have KDM and RDM activities. Whether or not JMJD6 is truly a bifunctional RDM/5-hydroxylase [55], extensive work on microbial 2OG oxygenases involved in secondary metabolite biosynthesis has revealed that such flexibility in substrate/product selectivity can and does occur in cells, so the potential for unanticipated reactivities of human (JmjC) 2OG oxygenases should not be excluded [3,56]. In the case of human 2OG oxygenases, the tendency to accept multiple substrates is probably best exemplified in the case of FIH. FIH was initially validated to catalyse the C-3 asparaginyl-hydroxylation of the C-terminal transcriptional activator domain of HIF-α [6,15]. Subsequently, FIH was shown to catalyse hydroxylation not only of asparaginyl, but also of aspartyl- and histidinyl-residues in cells on multiple members of the ARD family [31], which have roles including in, e.g. cell structure and the inflammatory response. In isolated form, the residue selectivity of FIH has been shown to be even broader, i.e. where it can accept serine and even hydrophobic residues [57].

**Figure 3.**
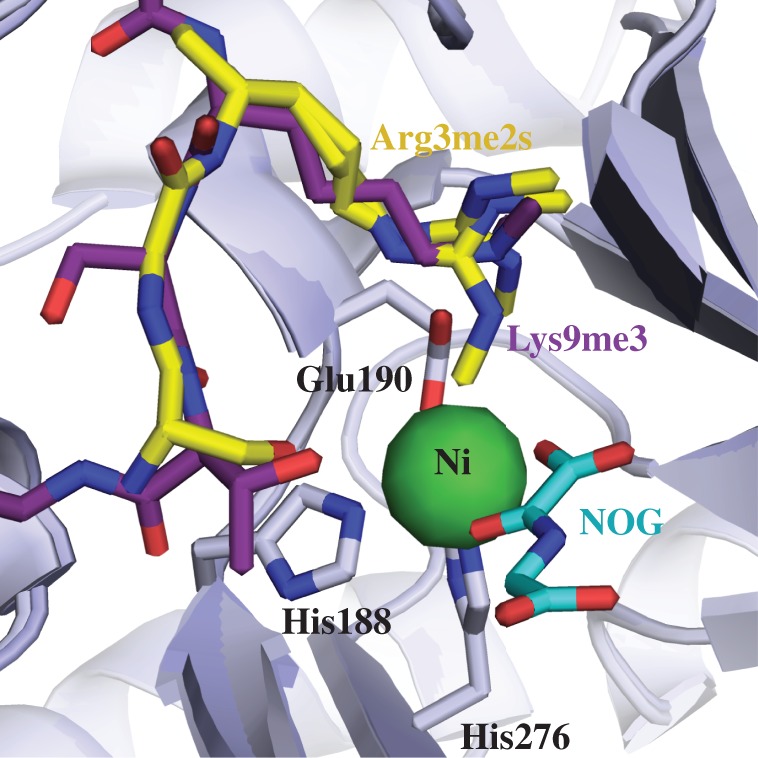
View from overlaid X-ray crystal structures of KDM4A complexed with nickel (substituting for iron), *N-*oxalylglycine (substituting for 2OG) and H4(1-15)R3me2s (yellow, PDB ID 2OX0) or H3(7-14)K9me3 (purple, PDB ID 5FWE) substrates showing similar binding mode for the *N*-methylated arginyl- and lysyl-residues at the KDM4A active site.

There is thus considerable interest in developing ‘substrate selective’ 2OG oxygenase inhibitors and, potentially, activators. Our results suggest that this may be difficult for the KDM and RDM activities of the JmjC demethylases with inhibitors that bind to the active site metal. As demonstrated in X-ray crystal structures (figure 3), both the *N*-methylated arginyl- and lysyl-residues (from two different peptides) bind the KDM4A active site with a similar orientation at a location separate to where *N-*oxalylglycine, or any other 2OG competitor, might bind; this further supports the findings that RDM activity is inhibited in a similar way to KDM inhibition by this class of inhibitors. An alternative approach would be to develop ‘allosteric’ inhibitors that disrupt binding of a particular type of substrate (e.g. to generate selective inhibitors for either KDM or RDM). As a step towards this, we have been working to develop substrate-competitive inhibitors and have recently reported on cyclic peptides that potently and selectively inhibit JmjC KDM4A, B and C [58].

Alternatively, it is possible that the non-catalytic domains of the JmjC demethylases could be targeted. The JmjC demethylases are much more than ‘mere’ catalysts, with most of them having additional domains to the JmjC/JmjN domains (with KDM4D, E and F being exceptions). The biological roles of these domains are still being explored; in some cases, they can regulate the JmjC KDM activity, as clearly exemplified in the case of KDM PHD finger protein 8 (PHF8), where its PHD domain located close to the *N*-terminus of the JmjC domain binds to H3K4me3 promoting catalysis at H3K9me2 [59,60]. Thus, at least in some cases, there is potential for the development of small molecules that bind to the PHD domain to inhibit H3K9me2 KDM activity. It is possible that non-catalytic domains of the JmjC demethylases, possibly in conjunction with interacting proteins and nucleic acids, guide substrate selectivity both towards specific protein sequences and the type of reaction catalysed (e.g. KDM versus RDM activity). To date, little has been reported on the development of ‘inhibitors’ binding to the non-catalytic domains of the JmjC demethylases [2,34,61] and although there are challenges, particularly with respect to biologically relevant assays, this is an objective of current work in our laboratory.

## 4. Material and methods

### (a) Assay reagents

Recombinant KDM4E(1-377) was produced in *E. coli* BL21(DE3)-R3 containing the pRARE2 plasmid and purified (to greater than 90% purity by SDS-PAGE analysis) as described [37]. Unless otherwise specified, reagents were from Sigma-Aldrich. All assays were performed in 50 mM HEPES (pH7.5) buffer. All peptides were prepared as C-terminal amides. H3(1-15)R2me2a (full sequence: AR(me2a)TKQTARKSTGGKA-NH_2_) was synthesized by Peptide Protein Research, while H3(1-15)K9me2 (full sequence: ARTKQTARK(me2)STGGKA-NH2) was synthesized by GL Biochem.

### (b) MALDI-TOF-mass spectrometry (MS)-based assays

Time-course assays: Four different enzyme mixes containing KDM4E at 2, 1, 0.5 or 0.25 µM were tested with both the H3(1-15)K9me2 and H3(1-15)R2me2a peptides, and demethylation activities were monitored over time. KDM4E was allowed to equilibrate to room temperature for 10 min; the substrate/cofactor mixture was then added to initiate the reaction. Final concentrations in 10 µl reaction volume: 10 µM peptide, 10 µM ammonium iron(II) sulfate hexahydrate ((NH_4_)_2_Fe(SO_4_)_2_), 100 µM sodium l*-*ascorbate, 10 µM 2-oxoglutaric acid (2OG), as described in [19]. The 2OG concentration used was at approximately the *K*_M_ value for KDM4E (electronic supplementary material, table S1). Reactions were quenched with 1:1 (v/v) aqueous methanol.

On a 96-spot MALDI plate, samples were mixed in a 1:1 ratio (v/v) with α-cyano-4-hydroxycinnamic acid (CHCA) dissolved in 50% acetonitrile, 0.01% (v/v) aqueous trifluoroacetic acid (CF_3_CO_2_H). The dried spots were analysed using a MALDI-TOF MS (Micro MX, Waters, UK) machine in the positive ion reflectron mode; flight tube voltage 12 000 V; reflectron voltage 5200 V. Spectral analysis was carried out using MassLynx 4.0 (Waters).

The relative peak intensities (RPI) of each methylation state were used to determine the percentage activity of the enzyme. All values were normalized using the percentage demethylation obtained from the first time point.

### (c) High-throughput liquid chromatography mass spectrometry (LC-MS) assays [62]

Assays were performed in 384-well polypropylene v-bottomed plates (Greiner Bio One). 2,4,PDCA, NOG, Deferoxamine, EDTA, KDOAM25, ML324, JIB-04, succinic acid disodium salt hexahydrate (succinate), fumaric acid (fumarate), l*-*2-hydroxyglutarate (HG), d*-*2HG, nickel (II) sulfate heptahydrate (Ni(II)) and cobalt (II) chloride hexahydrate (Co(II) were from Sigma-Aldrich, IOX1 was from Stratech Scientific, CPI 455 from Axon Medchem and KDM5-C49 and SD70 were from Xcess Biosciences. Structures of the inhibitors are defined in electronic supplementary material, figure S2.

Time-course assays: 1 and 0.25 µM (final concentrations in 50 µl reaction volume) enzyme mixes containing KDM4E were used with H3(1-15)R2me2a and the H3(1-15)K9me2, respectively; activity was monitored over time. KDM4E was incubated for 10 min with 1% (v/v) DMSO (dimethylsulfoxide) before addition of the cofactor/substrate mixture to initiate reaction. Final concentrations in 50 µl reaction volume: 5 µM respective peptide, 10 µM (NH_4_)_2_Fe(SO_4_)_2_, 100 µM sodium l*-*ascorbate, 10 µM 2OG. The reactions were quenched with 1% (v/v) aqueous formic acid (HCO_2_H). Assays were carried out with two analytical repeats.

Inhibition assays: compound dispensing (500 nl) was carried out using an ECHO 550 Acoustic dispenser (Labcyte). For each compound 12 different concentrations were prepared. Compound concentrations were decreased by a dilution factor of two between each point. Negative controls (no enzyme activity) and positive controls (maximum enzyme activity) all contained 500 nl DMSO without inhibitor.

The KDM4E enzyme mixture (24.5 µl) was transferred into each well containing 0.5 µl of the compound in DMSO and incubated for 10 min. The reaction was initiated upon addition of 25 µl of the substrate/cofactor mixture. Final concentrations in a 50 µl reaction volume were: 0.25 µM KDM4E (for KDM activity) or 1 µM KDM4E (for RDM activity), the compound at respective concentration, 1% (v/v) aqueous DMSO, 5 µM respective peptide, 10 µM (NH_4_)_2_Fe(SO_4_)_2_, 100 µM sodium l*-*ascorbate and 10 µM 2OG. The reaction was quenched with aqueous 1% (v/v) HCO_2_H after 15 min for KDM activity and 25 min for RDM activity.

The reactions were then transferred to a RapidFire RF360 high-throughput sampling robot connected to an Agilent 6530 Accurate-Mass Quadrupole time-of-flight (Q-TOF) mass spectrometer operated in the positive ion mode (Agilent, Wakefield, MA, USA). Prior to MS analysis, the samples were aspirated under vacuum for 400 ms then loaded onto a C-18 solid phase extraction (SPE) cartridge to remove buffer salts from the samples. This was achieved by washing the cartridge with 0.1% (v/v) HCO_2_H in water at a flow rate of 1.5 ml min^−1^ for 4.5 s and followed with an elution step (85% acetonitrile, 15% deionized water, 0.1% formic acid). The samples were loaded onto the MS with at a flow rate of 1.25 ml min^−1^ for 4.5 s. The cartridge was re-equilibrated with water for 500 ms.

For both KDM and RDM assays, the +5 charge state of the dimethylated substrate and the mono-methylated product were used to extract ion chromatogram data. Using RapidFire Integrator software (Agilent, Wakefield, MA, USA), the peak area data were integrated and produced the relative intensities at each point. The percentage activity was then calculated. The assays were repeated two or three times; and, in each case, this was carried out with three analytical repeats.

All data points were combined, producing six or nine dose response datasets and these were analysed collectively using GraphPad Prism 5. A non-linear curve with variable slope (the four-parameter logistic function) was applied to generate the best-fit LogIC_50_ values and the LogIC_50_ standard error mean (s.e.m.). pIC_50_ ± s.e.m. values were calculated using the equation, pIC_50_ = –Log (IC_50_/M).

## Supplementary Material

Figures S1 and S2; Table S1

## Data accessibility

The datasets supporting this article have been uploaded as part of the electronic supplementary material.
