# Supplementary material for: Inhibitors of both the N-methyl lysyl- and arginyl-demethylase activities of the JmjC oxygenases
Source: Philos Trans R Soc Lond B Biol Sci. 2018 Apr 23;373(1748):20170071. doi: 10.1098/rstb.2017.0071 (PMC5915715; doi:10.1098/rstb.2017.0071)
Supplement: Figures S1 and S2; Table S1 [file rstb20170071supp1.pdf]

Supplementary Material

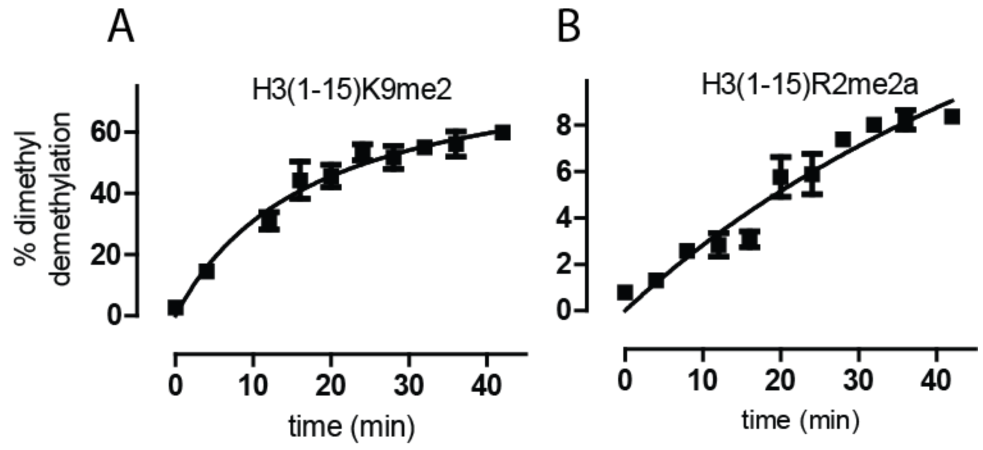

Figure S1: Time course assays using LC-MS showing the extent of KDM (A) or RDM (B) demethylation. KDM (A) and RDM (B) activities are represented as percentage of the dimethylated peptide demethylated (average ± standard error of the mean (SEM), n=2 analytical repeats*). Conditions: 5µM respective peptide, 10µM Fe(II), 100µM sodium L-ascorbate, 10µM 2OG, 1% DMSO. A: 0.25 µM KDM4E, B: 1 µM KDM4E.* Due to different specific activities for KDM and RDM activities (A: 13 nmoles/min/mg, B: 0.3 nmoles/min/mg), different endpoints were used; A: 15 minutes, B: 25 minutes.

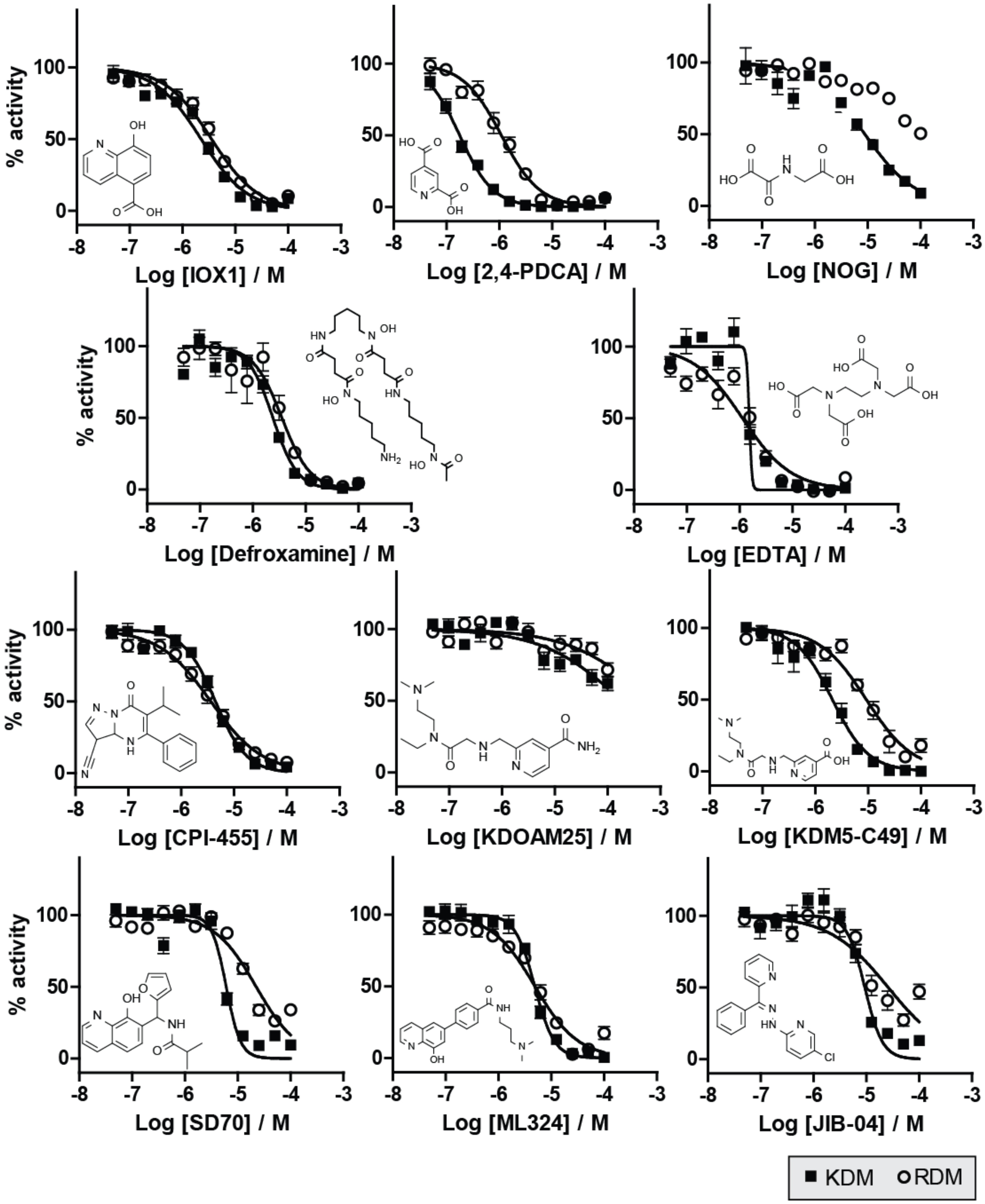

Figure S2. Comparison of the KDM and RDM activities of KDM4E. IC_{50} determinations were performed using LC-MS (See Materials and Methods). Error bars represent SEM for n = 3 assay repeats, each with n = 3 with respect to analytical repeats, except for Defroxamine and EDTA which had n = 2 assays repeats each with n = 3 analytical repeats. *Conditions: 5 µM H3(1-15)K9me2/H3(1-15)R2me2a with 0.25 µM/1µM KDM4E, respectively, 10 µM (NH4)2Fe(SO4)2, 100 µM sodium L-ascorbate, 10 µM 2OG, with t = 15/25 mins, for KDM or RDM activities, respectively).* Refer to Table 1 for pIC_{50} values.

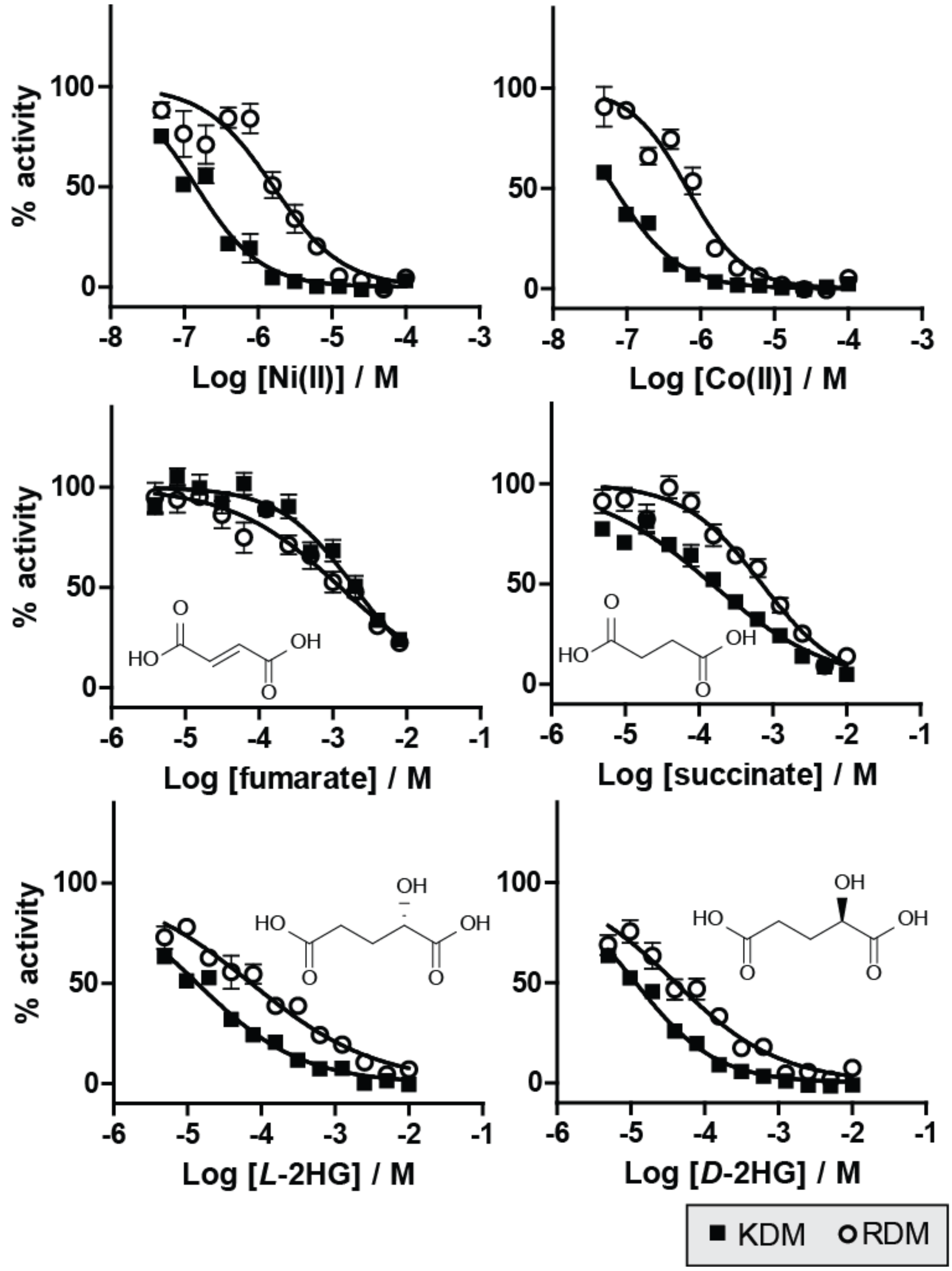

Figure S2 (cont). Comparison of the KDM and RDM activities of KDM4E. IC_{50} determinations were performed using LC-MS (See Materials and Methods). Error bars represent SEM for n = 2 assay repeats each with n=3 analytical repeats. *Conditions: 5 µM H3(1-15)K9me2/H3(1-15)R2me2a with 0.25 µM/1µM KDM4E, respectively, 10 µM $(NH_4)_2Fe(SO_4)_2$, 100 µM sodium L-ascorbate, 10 µM 2OG, with t = 15/25 mins, for KDM or RDM activities, respectively).* Refer to Table 1 for pIC_{50} values.

Substrate	K_M/ µM	k_{cat} x 10^{-3} / s^{-1}	k_{cat}/ K_M x 10^{-6}
2OG [(1)]	14.0 ± 1.8	0.087 ± 0.003	6.2
H3(1-15)K9me3 [(2)]	17.8 ± 4.0	170.7 ± 15.2	9,575
H3(1-15)K9me2 [(3)]	32.3 ± 7.4	70.0 ± 4.0	2167.2
H3(1-15)R2me2a [(2)]	86.9 ± 16.5	9.9 ± 1.0	114
H4(1-15)R3me2a [(2)]	121.5 ± 10.1	2.1 ± 0.1	17.7

Table S1: Kinetic parameters (K_M, k_{cat} and k_{cat}/ K_M) for substrates and 2OG performed using the formaldehyde dehydrogenase based coupled assay measuring formaldehyde production as described in (4). Data show mean ± SEM (n = 3).

References

1. Sakurai M, *et al.* A miniaturized screen for inhibitors of Jumonji histone demethylases. Mol Biosyst. 2010;**6**:357-364.
2. Walport LJ, *et al.* Arginine demethylation is catalysed by a subset of JmjC histone lysine demethylases. Nat Commun. 2016;**7**:11974.
3. Rose NR. Structural, mechanistic and inhibition studies on the histone lysine demethylases: Thesis (D.Phil.)--University of Oxford.; 2009.
4. Rose NR, *et al.* Inhibitor Scaffolds for 2-Oxoglutarate-Dependent Histone Lysine Demethylases. J Med Chem. 2008;**51**:7053-7056.
